# Fine Mapping of *CsVYL*, Conferring Virescent Leaf Through the Regulation of Chloroplast Development in Cucumber

**DOI:** 10.3389/fpls.2018.00432

**Published:** 2018-04-06

**Authors:** Mengfei Song, Qingzhen Wei, Jing Wang, Wenyuan Fu, Xiaodong Qin, Xiumei Lu, Feng Cheng, Kang Yang, Lu Zhang, Xiaqing Yu, Ji Li, Jinfeng Chen, Qunfeng Lou

**Affiliations:** State Key Laboratory of Crop Genetics and Germplasm Enhancement, College of Horticulture, Nanjing Agricultural University, Nanjing, China

**Keywords:** *Cucumis sativus* L., leaf color mutant, virescent, chloroplast function, DnaJ proteins

## Abstract

Leaf color mutants in higher plants are ideal materials for investigating the structure and function of photosynthetic system. In this study, we identified a cucumber *vyl* (virescent-yellow leaf) mutant in the mutant library, which exhibited reduced pigment contents and delayed chloroplast development process. F_2_ and BC_1_ populations were constructed from the cross between *vyl* mutant and cucumber inbred line ‘Hazerd’ to identify that the *vyl* trait is controlled by a simply recessive gene designated as *CsVYL.* The *CsVYL* gene was mapped to a 3.8 cM interval on chromosome 4 using these 80 F_2_ individuals and BSA (bulked segregation analysis) approach. Fine genetic map was conducted with 1542 F_2_ plants and narrowed down the *vyl* locus to an 86.3 kb genomic region, which contains a total of 11 genes. Sequence alignment between the wild type (WT) and *vyl* only identified one single nucleotide mutation (C→T) in the first exon of gene *Csa4G637110*, which encodes a DnaJ-like zinc finger protein. Gene Expression analysis confirmed the differences in transcription level of *Csa4G637110* between wild type and mutant plants. Map-based cloning of the *CsVYL* gene could accelerate the study of chloroplast development and chlorophyll synthesis of cucumber.

## Introduction

Leaf is a vital organ for plant photosynthesis that occurs mainly in chloroplasts. Chloroplast, a semi-autonomous organelle exits only in plant cells, can synthesize various metabolites that are essential for plant growth and development, and maintenance of green leaf color (Moreira et al., 2000). Leaf color mutants are formed due to silencing or inactivation of genes controlling chlorophyll biosynthesis and chloroplast development, which directly or indirectly affects chlorophyll synthesis and degradation, ultimately leading to leaf color variation (Motohashi et al., 2003). Therefore, leaf color mutants are the ideal materials to study the mechanism of plant photosynthesis, chloroplast ultrastructure, chlorophyll biosynthetic pathway, expression and regulation of related genes (Stern et al., 2004). So far, leaf color mutants have been obtained in rice (Zhang et al., 2006), wheat (Falbel et al., 1996), maize (Lonosky et al., 2004), soybean (Zhang et al., 2011), cotton (Karaca et al., 2004), carrot (Nothnagel and Straka, 2003) and cucumber (Gao et al., 2016). Numerous studies have been reported in relative genetic, physiological and molecular mechanism research.

Virescent, which shows light yellow cotyledons or true leaf and turns green gradually during leaf development, is a special and important mutation type for understanding the mechanism of chloroplast formation and most of virescent mutant are thermo- or light-sensitive (Archer and Bonnett, 1987; Yoo et al., 2009). A few virescent mutants have been reported in Arabidopsis (Koussevitzky et al., 2007), rice (Sugimoto et al., 2007), maize (Hopkins and Elfman, 1984) and other plants. Several genes have been positional cloned in these plants. In rice, Dong et al. (2013) used a temperature-insensitive and developmental stage-dependent virescent yellow leaf (*vyl*) mutant to clone the *vyl* gene related to plastidic caseinolytic protease. Xing et al. (2014) reported a virescent yellow-like (*vyl*) maize mutant that exhibited young yellow leaf phenotype but gradually recovered, and identified a gene *Chr.9_ClpP5* as the candidate gene, which is one of the proteolytic subunits of the chloroplast Clp protease complex. In cucumber, Miao et al. (2016) used a virescent leaf mutant 9110Gt showing light yellow cotyledons and first true leaf to detect a single recessive gene (*v-1*) controlled the virescent leaf trait, and identified a most likely candidate gene for the *v-1* by high-resolution genetic mapping.

DnaJ proteins are important molecular chaperones in the cell and play significant roles in protein folding, assembly, localization, transportation and denatured protein renaturation or degradation (Hartl, 1996; Walsh et al., 2004). A 41 kDa heat shock protein DnaJ was first found in *Escherichia coli* (Georgopoulos et al., 1980), and subsequent studies have shown that DnaJ proteins are ubiquitously found in humans, animals, plants and fungi (Zhu et al., 1993; Terada et al., 1997; Miernyk, 2001). Canonical DnaJ proteins consist of a J-domain, a glycine/phenylalanine (G/F) domain, a zinc finger domain (included four repeats of the CxxCxGxG sequence) and a C-terminal domain (Walsh et al., 2004). Different from the DnaJ proteins, DnaJ-like zinc finger proteins lack of characteristic J-domain and G/F domain of typical DnaJ proteins structure, and only have one zinc finger domain in the C-terminal (CxxCxGxG sequence repeat in 2–4 times) (Lu et al., 2006). Previous studies have shown that most of the DnaJ-like zinc finger proteins have important regulatory functions for plastid development, photosynthesis, chloroplast movement, plant resistance and defense response (Shimada et al., 2007; Fristedt et al., 2014; Muñoz-Nortes et al., 2017).

Cucumber, *Cucumis sativus* L. (2n = 2x = 14) is an important vegetable crop worldwide. Although many studies of leaf color mutants have been reported in cucumber (Pierce and Wehner, 1990), little work has been performed on map-based cloning of mutant gene or explaining its regulation mechanism. In this study, a novel light-sensitive virescent-yellow leaf cucumber mutant, *vyl*, was identified in an ethyl methanesulfonate (EMS)-mutagenized Changchunmici (‘CCMC’, north China type) cucumber population. The mutant exhibited virescent yellow leaf at emergence with decreased chlorophyll accumulation and impaired chloroplast structure. Yellow leaf would turn green during development or under low light condition. The present study describes the results on phenotypic, physiological characterization and genetic of the cucumber *vyl* mutant and fine mapping of the candidate gene, *CsVYL*, which encodes a DnaJ-like zinc finger protein. These findings may facilitate the understanding of chloroplast development and chlorophyll synthesis in cucumber.

## Materials and Methods

### Plant Materials and Phenotypic Data Collection

The virescent yellow-leaf (*vyl*) mutant was isolated from an M_2_ family derived from an EMS-mutagenized cucumber ‘Changchunmici’ (CCMC, wild type, WT, a common inbred line with green leaf) population. Mutagenized plants of this second generation were self-pollinated for two generations to make the mutated gene homozygous, and then an F_2_ and BC_1_ populations were produced with the *vyl* (female) and green leaf cucumber inbred line ‘Hazerd’ (male) as the parents. The M_4_ generation, along with the F_1_, F_2_, and BC_1_ generations, were subsequently utilized to elucidate the inheritance pattern of *vyl*. A total of 80 individuals from F_2_ population were used for BSA analysis and then another 400 and 1065 individuals from F_2_ population were planted to fine map the *vyl* gene. All plants were grown in the greenhouse at Jiangpu Cucumber Research Station of Nanjing Agricultural University, Nanjing, China.

The leaf color of each plant was determined by visual inspection at 3–5 days after emergence. Chi Square Goodness of fit test was performed on phenotypic data to verify deviations from the expected 3:1 segregation in the F_2_ population or 1:1 segregation in the BC_1_ population.

### Measurement of Pigment Contents and Photosynthetic Parameters

Leaves of *vyl* mutant and wild type plants at four different leaf positions (leaf 1, 2, 3, and 4) were collected when the plants grow to the stage with 6–7 true leaves and used to determine chlorophyll content according to the method described by Arnon (1949) with some modifications. Chlorophyll a and chlorophyll b were calculated according to the formula:

$$\begin{array}{c} \text{Ca}\;\text{=}\;\text{13}.\text{95}\;\times \;\text{OD665}\;\text{−}\;\text{6}.\text{88}\;\times \;\text{OD649}\\ \text{Cb}\;\text{=}\;\text{24}.\text{96}\;\times \;\text{OD649}\;\text{−}\;\text{7}.\text{32}\;\times \;\text{OD665} \end{array}$$

Total carotenoid content of the leaf was measured as described by Soto-Zamora et al. (2005). The absorbance was determined in the spectrophotometer at 470 nm.

In addition, photosynthetic parameters including net photosynthetic rate (Pn), stomatal conductance (Gs), and intercellular CO_2_ concentration (Ci) of *vyl* mutant and wild type plants were measured in the field from 9:00 to 12:00 on the sunny day using the US-made LI-6400 portable photosynthesis measurement system. Chlorophyll fluorescence was measured using a MINI-PAM (Walz, Effeltrich, Germany). The maximum quantum efficiency of Photosystem II (PSII), F_v_/F_m_ = (F_m_–F_o_)/F_m_ (where F_o_ is the minimal and F_m_ the maximal fluorescence yield in dark-adapted leaves), was measured at the beginning of the day from leaves that were dark-adapted for 30 min in dark leaf clips DLC-8 (Walz, Effeltrich, Germany). The measurement was performed on the first leaf to the fourth leaf leaves on each plant in the same way. There individuals were measured on each repeat at four leaf positions and then the average was taken.

### Different Light Intensity Treatment

We found that *vyl* might be a light-sensitive mutant, because its leaf color changed as light intensity changed. To verify the correlation between *vyl* leaf color and light intensity, WT and *vyl* mutant were planted under three different levels of light intensity conditions, through the use of shading. Three light intensity treatments included full light (no shading, about 65000 LUX at the noon), 68% of full light, and 3% of full light. The corresponding light intensity was measured by the digital illuminometer Peakmeterm-S6612. The phenotype of *vyl* under different light intensity treatments was identified as well as its chlorophyll contents.

### Transmission Electron Microscopy

Leaf samples of wild type and *vyl* mutant were prepared for TEM from the 1st, 2nd, 3rd, and 4th leaf with the blade when the 4th leaf expanded fully. Leaf sections were vacuumized and fixed in 2.5% glutaraldehyde in a phosphate buffer at 4°C for 4 h. The samples were dehydrated in a graded ethanol series, and critical-point drying was done using liquid CO_2_ in a critical-point drier Bal Tec CPD 030 and 15 nm gold coated on aluminum stubs in a Sputte Coater Bal-Tec SCD 005. The samples were visualized with a Hitachi S-3500N scanning electron microscope.

### DNA Extraction and BSA

Young leaf samples of each individual were collected and frozen in liquid nitrogen and maintained at -80°C for further use in experiments. Genomic DNAs were extracted from leaves by the modified cetyltrimethylammonium bromide (CTAB) method of Murray and Thompson (1980). For the bulked segregant analysis (BSA), 10 individuals with virescent yellow color leaf and 10 with green color were randomly selected from the 80 plants of F_2_ population, and two DNA bulks were made by bulked equal quantities of DNA of the 10 individuals.

### Molecular Marker Development and Mapping Strategy

In initial mapping phase, 450 published SSR (simple sequence repeat) markers evenly distributed on 7 chromosomes were selected randomly from the SSR library of ‘9930’ (Ren et al., 2009) and ‘Gy14’ (Cavagnaro et al., 2010) to screen for polymorphisms between the two parents and two pools. Then polymorphism markers were used to identify the genotypes of each individual of 80 plants from the F_2_ mapping population and construct an initial genetic map. When no recombination events was observed between the flanking markers, the size of the mapping population was expanded to 480 plants for further testing (including the initial 80 plants). Additional SSR markers in the preliminary mapping region were developed by screening the relative sequences of ‘9930’ genome, which were available from the Cucurbit Genomics Database^1^, using the SSR Hunter 1.3 program.

A larger F_2_ population (*n* = 1545, including initial 480 plants) were applied to fine map the *CsVYL* locus. Two parents, *vyl* mutant and ‘Hazard,’ were re-sequenced by the Illumina Hi-Seq 4000 platform to develop more available molecular markers. Short reads obtained from two parents were aligned against the cucumber genome sequence (the ‘9930’ reference genome) to obtain the consensus sequence using BWA software (Huang et al., 2009; Langmead and Salzberg, 2012). Reads were aligned to ‘9930’ consensus sequence reads to call SNPs with SAM tools software (Li and Durbin, 2009). The correlated sequences of two mapping parents in the initial interval were used to develop Indel and CAPS molecular markers. Insertions and deletions (>3 bp) were screened using the SAM Tool program in this interval to design Indel markers by Primer 5.0. In addition, the SNPs were converted into CAPS (cleaved amplified polymorphic sequences) markers using SNP2CAPS program. Finally, polymorphic markers between two parents were further screened from the newly developed markers and used to identify recombinants in the 1545 plants of the F_2_ population for fine mapping the *CsVYL* locus. Primers of molecular makers used in fine mapping of *CsVYL* are listed in Supplementary Table 1.

The linkage map was constructed using Join Map 4.0 with a LOD threshold score of 3.0. The PCR reaction and gel electrophoresis was conducted as described in Li et al. (2011).

### Gene Annotation and Candidate Gene Identification

Candidate genes in target region were predicted using the online program FGENESH^2^ and the Cucumber Genome Database^3^ (Huang et al., 2009) and their annotations were obtained with the BLASTP tool from NCBI^4^. For the candidate gene identified, the DNA, cDNA and protein sequences alignments were performed using the software DNAMAN to detect variations between *vy1* and WT. And in order to verify the reliability of candidate gene, the DNA sequence of candidate gene were sequencing among 72 natural cucumber lines. Protein sequences of *CsVYL* and its homologous were downloaded from NCBI database. Primers used for cloning of candidate gene sequence are listed in Supplementary Table 2.

### Quantitative Reverse-Transcription PCR (qPCR) Analysis

Total RNA was extracted from the first (yellow stage) and fifth leaves (green stage) of CCMC (WT) and *vyl* (representing yellow and green stages) with RNAprep Plant Mini Kit (Tiangen, Beijing, China) following the manufacturer’s instructions. First-strand cDNA was synthesized using Prime Script 1st Strand cDNA Synthesis Kit (TaKaRa, Kyoto, Japan). To analyze gene expression, quantitative real-time PCR (qPCR) was carried out using the Real Master Mix Kit (SYBR Green, Tiangen). qRT-PCR and PCR amplification was quantified according to the manufacturer’s protocol. All experiments were performed with three biological replicates. The 2^-ΔΔC_T_^ method was used to analyze the relative mRNA expression level of *vyl*, and the values represented the n-fold difference relative to the gene expression of WT at the yellow stage. Primers used for qPCR are listed in Supplementary Table 3.

## Results

### The *vyl* Is a Development Dependent and Light Sensitive Mutant

**FIGURE 1:**
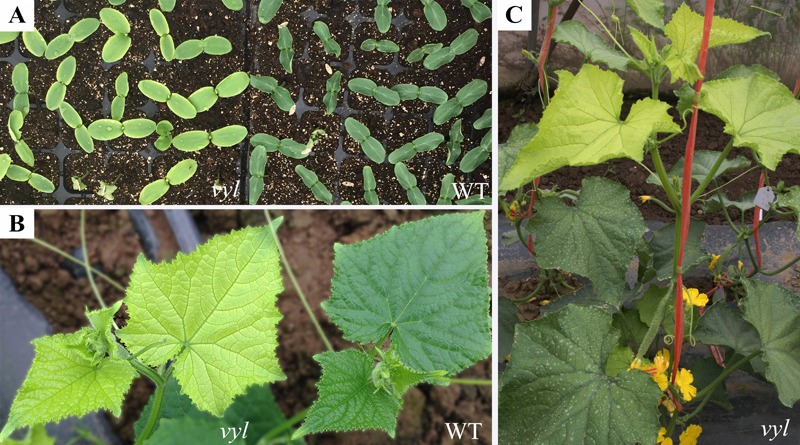
Phenotypic analysis of the *vyl* mutant. **(A)** Cotyledons color of WT and *vyl*; **(B)** Detailed observations of leaf color of WT and *vyl* grown in field; **(C)** Leaf color of *vyl* at plant adult stage.

A mutant with virescent yellow-leaf (*vyl*) was identified in an M_2_ generation from EMS-mutagenized ‘CCMC’ population which exhibited virescent yellow leaf at emergence and then gradually recovered (**Figures 1A,C**). Different from leaf color mutant reported previously (Miao et al., 2016), the mutant studied in this work exhibited virescent yellow leaf only in true leaves whereas the leaf vein remains green (**Figure 1B**). At maturity, the *vyl* mutant could normally blossom, bear fruits and produce seeds (**Figure 1C**).

**FIGURE 2:**
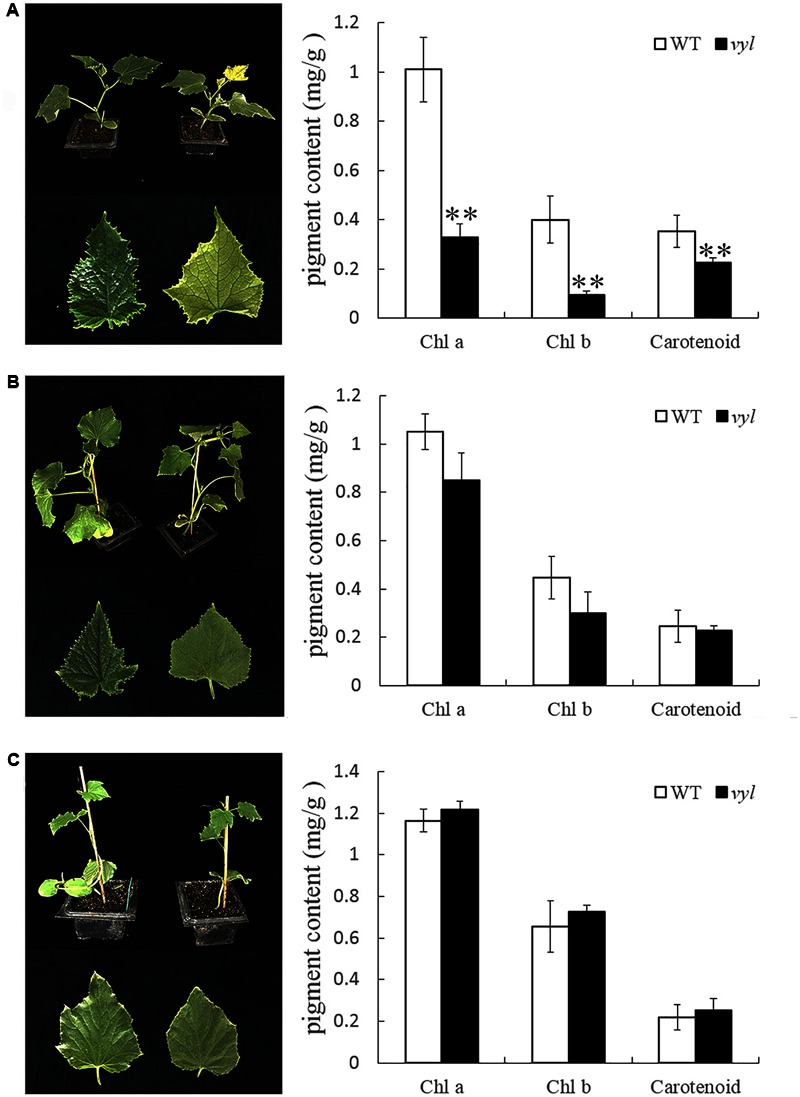
The leaf color phenotype and pigment contents of *vyl* and WT under different light intensity treatments. L1 of *vyl* and WT planted under 100% **(A)**, 68% **(B)** and 3% **(C)** of full light conditions was used for leaf color observation and Chl measurement. Values are the mean ± SD, ^∗∗^*P* < 0.01.

To investigate whether the photosynthetic apparatus was affected in *vyl* mutants, we compared the content of some key photosynthetic pigments between *vyl* and WT plants (**Table 1**). The contents of chloroplyll a, chloroplyll b and carotenoid were basically consistent in WT at different leaf positions, whereas the contents of the photosynthetic pigments in *vyl* mutant were significantly decreased compared to those in WT at early leaf positions (the first to the third leaf from the top). When the leaf grows, leaf color turned green gradually and photosynthetic pigments increased to reach the level in WT after the fourth leaf. To verify whether *vyl* is a light-sensitive mutant, WT and *vyl* were planted under different light intensity conditions and the change of leaf color and Chl contents of *vyl* was examined. As shown in **Figure 2A**, under 100% full light condition, compared with the WT plants, the *vyl* mutant exhibited extreme chlorosis and its Chl contents were lower than those in WT. As the *vyl* mutant grew under reduced light intensity condition, leaf color of *vyl* showed a greening trend, and Chl contents also increased (**Figures 2B,C**). Notably, under extreme low light condition (about 3% full light), the *vyl* mutant showed the same leaf color phenotype and Chl contents as WT under the same conditions (**Figure 2C**). These results indicated that virescent-yellow leaf in this mutant is a light sensitive trait.

**Table 1:** Comparison of pigment content between *vyl* and the wild type at different leaf position.

Leaf	Materials	Chloroplyll	Chloroplyll	Carotenoid
position		a (mg/g)	b (mg/g)	(mg/g)
L1	Wild type	1.1 ± 0.12	0.46 ± 0.10	0.44 ± 0.07
	*vyl*	0.40 ± 0.08**	0.14 ± 0.03**	0.20 ± 0.05**
L2	Wild type	1.14 ± 0.09	0.46 ± 0.09	0.44 ± 0.08
	*vyl*	0.54 ± 0.05**	0.20 ± 0.05**	0.24 ± 0.07**
L3	Wild type	1.14 ± 0.18	0.44 ± 0.06	0.42 ± 0.04
	*vyl*	0.66 ± 0.09**	0.24 ± 0.04**	0.26 ± 0.04**
L4	Wild type	1.14 ± 0.12	0.44 ± 0.04	0.42 ± 0.05
	*vyl*	0.92 ± 0.09	0.36 ± 0.02**	0.34 ± 0.03

*^∗∗^Means significant differences at 0.01 level. L1, L2, L3, L4 indicate different leaf position, for example, L1 means the first leaf below the plant top.*

### The *vyl* Has Defective Chloroplast Development and Reduced Photosynthetic Capacity at Early Leaf Stage

**FIGURE 3:**
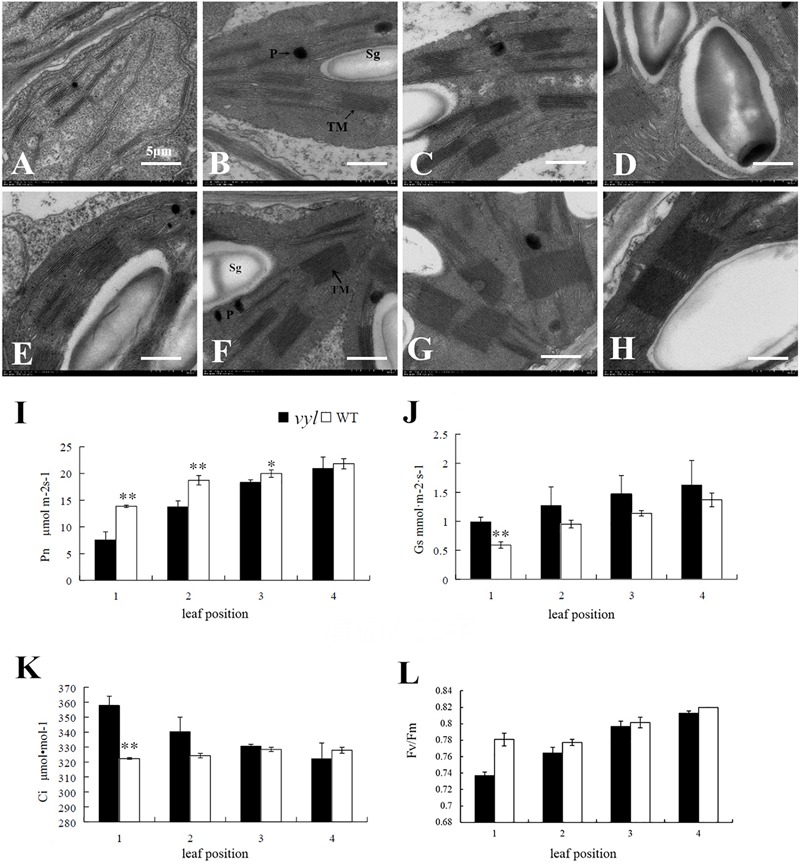
Chloroplast structure and photosynthesis parameters of *vyl* and WT at different leaf position. Electron microscopy of chloroplasts ultrastructures in L1-L4 of *vyl* **(A–D)** and WT **(E–H)**. TM, thylakoid membrane; Sg, starch grain; P, plastoglobulus. Scale bar = 500 nm. **(I)** Net photosynthetic rate (P_n_); **(J)** Stomatal conductance (Gs); **(K)** Intercellular CO_2_ concentration (Ci); **(L)** Photosystem II (F_v_/F_m_); Values are the mean ± SD, and ^∗^(*P* < 0.05) and ^∗∗^(*P* < 0.01) indicate significant difference between the treatments.

Mesophyll cells of the first to the fourth leaf (from the plant top) from *vyl* mutant and WT were examined by TEM. The impaired chloroplast development with various degrees in the mutant was observed, especially in the first to the third leaf, and recovered to the similar level as WT eventually, after the fourth leaf (**Figures 3A–D**), whereas the WT had a morphologically well-structured thylakoid system at each leaf position (**Figures 3E–H**). Notably, membrane system was outspread and the thylakoids were not stacked into grana at the first leaf in *vyl*. As photosynthetic assimilation products, starch grains were hardly observed at the first leaf in *vyl* (**Figure 3A**), which was clearly visible in WT (**Figure 3E**). This result also indicated the difference of photosynthetic function between *vyl* mutant and WT. At the second leaf in the mutant (**Figure 3B**), stacked grana and starch grain were observed. As the leaf turned green at the third leaf (**Figure 3C**), more stacked grana and starch grain were observed in *vyl*. At the fourth leaf in *vyl* (**Figure 3D**), there was more starch grain and the stacked grana exhibiting well developed lamellar structures, similar to that in WT (**Figure 3H**).

Several key photosynthetic parameters were compared at different leaf positions in *vyl* mutant and WT plants (**Figure 3**). Young leaves of *vyl* mutant showed significantly lower P_n_ than WT plants (**Figure 3I**). Because both ‘stomatal’ and ‘non-stomatal’ components can contribute to the decrease of P_n_ (Quick et al., 1992), and the value of sub-stomatal CO_2_ concentration (Ci) can be used to distinguish the two components (Farquar and Sharkey, 1982). Higher Gs and Ci suggest that the decrease of P_n_ in *vyl* mutant young leaves was caused by non-stomatal component (**Figures 3J,K**). As leaf matured, *vyl* mutant and WT plants displayed similar photosynthesis, indicating the recovery of photosynthesis (**Figure 3**). Similar trend was also observed in the maximum quantum efficiency of PSII (F_v_/F_m_). F_v_/F_m_ was lower in the young leaf of *vyl* mutant than in WT plants, suggests that the photosystem of *vyl* mutant was undeveloped compared to the contemporaneous WT plants (**Figure 3L**).

### Genetic Mapping of *CsVYL* Gene Using BSA Method

To determine the inheritance patterns of virescent yellow-leaf trait, the *vyl* was crossed with ‘Hazerd’ (green leaf) to generate F_2_ and BC_1_ populations. The F_1_ individuals have green leaf, and among the 80 plants of F_2_ population, there are 58 green leaf individuals and 22 *vyl* individuals, corresponding with the 3:1 ratio of Mendel’s law of segregation (χ^2^ = 0.27, *p* = 0.61). For the 100 plants derived from backcross with the *vyl* parent, 53 had green leaf and 47 were *vyl* leaf, conforming to the 1:1 ratio of Mendel’s law of segregation (χ^2^ = 0.36, *p* = 0.55). The segregation ratios obtained above supported that a single recessive gene, *CsVYL*, controls the virescent yellow leaf trait.

**FIGURE 4:**
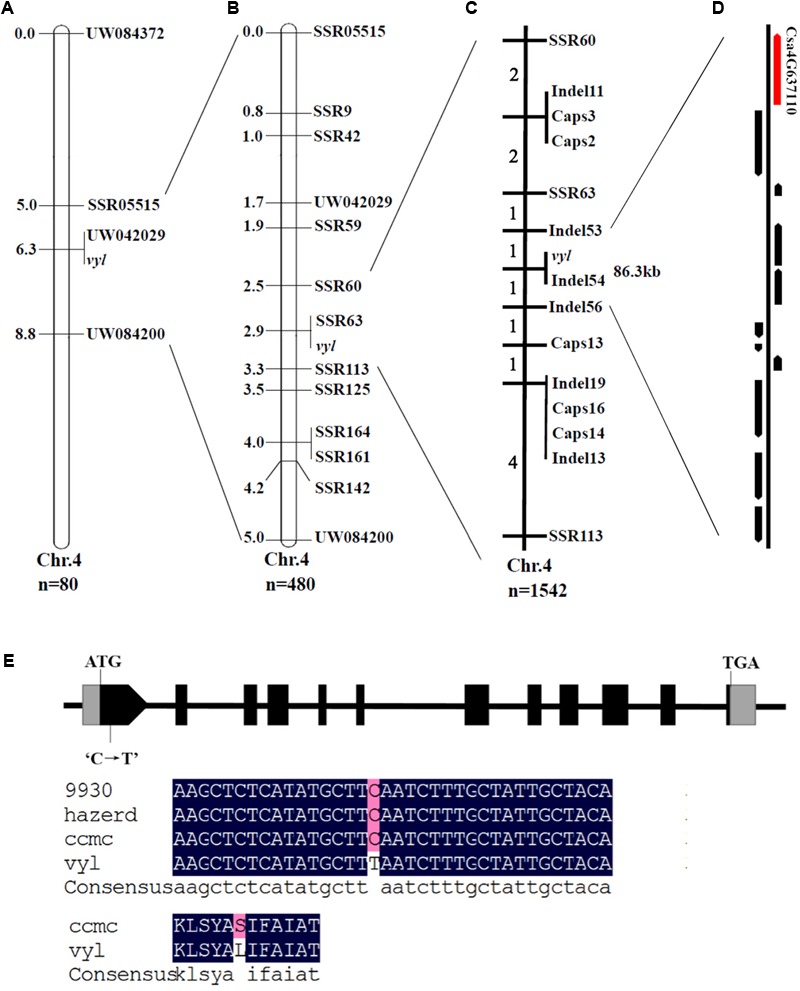
Positional cloning and sequence alignment of the *CsVYL* gene. **(A)** Preliminary mapping with BSA based on 80 plants from the F_2_ population placed *CsVYL* locus on chromosome 4 at a 3.8 cM region between UW804200 and SSR05515. Numbers on chromosome left are genetic distance (cM); **(B)** Further genetic position analysis with 480 F_2_ individuals delimited the *CsVYL* locus at a 0.8 cM region; **(C)** Fine mapping of the *CsVYL* locus with 1542 individuals. The *CsVYL* gene was narrowed down to an 86.3 kb genetic region. Numbers on chromosome left are recombinants of each interval; **(D)** 11 annotated genes were predicted in the 86.3 kb region. *Csa4G637110* is shown as the red arrow. **(E)** Gene and protein sequence alignment of *CsVYL* gene in 9930, Hazerd, ccmc and *vyl* Gray boxes, black boxes and lines between them indicate 5′ and 3′UTR, exons and introns respectively.

Genetic mapping of the *CsVYL* gene was performed using bulked segregation analysis approach. First, among the 450 SSR markers evenly distributed on seven cucumber chromosomes, 84 polymorphic SSR markers (18.7%) between the parents, *vyl* and ‘Hazerd,’ were screened. Then using two DNA sample pools from *vyl* mutant and green leaf individuals of the F_2_ population as templates, the 84 markers were used to determine linkage relationship between the markers and *vyl* phenotype. Four SSR markers (UW084200, UW042029, SSR05515 and UW084372) were observed to be linked with the *vyl* phenotype. These four SSR markers were used to genotype 80 F_2_ individuals and *CsVYL* locus was located within an approximately 3.8 cM interval between UW084200 and SSR05515, co-segregating with UW042029 on distal end of the long arm of chromosome 4 (**Figure 4A**). Since no recombination events was observed between the flanking markers, the size of mapping population was expanded to 480 plants for further testing (including the initial 80 plants). To narrow down the target region, the genomic sequence of ‘9930’ in the target interval was used to develop new SSR markers. Totally 184 pairs of SSR makers were developed in this region and 10 polymorphic markers (5.4%) were detected. All polymorphic markers, two flanking markers, UW084200 and SSR05515 and one co-segregating marker (UW042029) were used to construct a local genetic map of the *CsVYL* locus using 480 F_2_ individuals. Two closest markers, SSR60 and SSR113, flanked the *CsVYL* locus at a 0.8 cM interval (∼467 kb), and a co-segregating marker, SSR63 was found (**Figure 4B**).

### Fine Mapping Using Genome Sequence Data

Since additional effort of exploring markers in the region flanked by SSR60 and SSR113 couldn’t develop new markers that were closer to the target gene, we conducted high-depth re-sequencing of two mapping parents, *vyl* mutant and ‘Hazerd,’ to develop Indel and SNP markers (The Illumina sequence raw data have been submitted to NCBI SRA database, accession number SRP134028, and re-sequencing data is listed in Supplementary Table 4). Indels that varied > 3 bp in length were selected to develop Indel markers. Totally 74 Indel markers were developed, among which, 6 markers that showed obvious polymorphism between two parents were used to fine map the *CsVYL* locus. In addition, 35 CAPS makers were developed by screening SNP between the target region and 5 of them were polymorphic between two parents. Using flanking markers, SSR60 and SSR113, 13 recombinant plants were selected from 1542 plants from the F_2_ population. Eleven newly developed markers, two flanking markers and one co-segregating marker were subsequently used to map the *CsVYL* locus with the 13 recombinant plants. Finally, fine genetic mapping placed the *CsVYL* locus in the genomic region flanked by markers Indel53 and Indel56, which was approximately 86.3 kb in cucumber reference genome (**Figure 4C**).

### Candidate Gene Identification

In the 86.3 kb region, 11 genes were annotated using the online program FGENESH and ‘9930’ genome sequence data (**Figure 4D**). The information and predicted functions of the 11 genes were presented in **Table 2**. Coding sequences of these genes were sequenced and aligned between WT and *vyl* mutant, and a single nucleotide mutation occurred in gene *Csa4G637110* was found, whereas no differences were found in other 10 genes. Besides, the single nucleotide mutation of the candidate gene existed only in the *vyl* mutant, but not in other 72 natural populations (Supplementary Figure 1). Annotation of *Csa4G637110* revealed that there were 31 exons and 11 introns in this gene. The genomic sequence of this gene from WT and *vy1* was cloned, and the full length of this gene is 8149 bp in ‘9930’ reference genome. Sequence alignment between WT and *vy1* indicated that a single nucleotide mutation (C→T) occurred in the first exon of the *Csa4G637110* gene, which resulted in an amino acid change of glutamic to lysine acid (**Figure 4E** and Supplementary Figure 2).

**Table 2:** Predicted genes in the 86.3 kb region.

ORF No.	Gene ID	Position in 9930 genome	Gene annotation
1	*Csa4G637110*	20695774..20703922	DnaJ-like zinc finger protein
2	*Csa4G637120*	20705767..20713243	Bifunctional methylthioribulose-1-phosphate dehydratase
3	*Csa4G637130*	20716210..20717384	VQ motif-containing protein
4	*Csa4G637140*	20730318..20734988	Dynamin-related protein
5	*Csa4G637150*	20735565..20739818	Proton pump-interactor
6	*Csa4G637160*	20744740..20746545	Xylosyltransferase
7	*Csa4G637170*	20746608..20747443	Acetylglucosaminyltransferase
8	*Csa4G637180*	20748636..20750371	Pentatricopeptide repeat-containing protein
9	*Csa4G637680*	20751673..20758425	SPA1-related protein
10	*Csa4G637690*	20766468..20776209	Uncharacterized protein
11	*Csa4G637700*	20780049..20785461	Ubiquitin-conjugating enzyme

*ID number given by cmb (http://cmb.bnu.edu.cn/Cucumis_sativus_v20).*

Protein sequences of the encoded protein and other 9 homologs were aligned. Detail alignment showed that all proteins have an incomplete zinc finger domain with two C-x(2)-C-x-G-x-G repeats and incomplete C-x(2)-C-x-G or C-x(2)-C repeats, different from the typical zinc finger domain of DnaJ protein which has four C-x(2)-C-x-G-x-G repeats (Supplementary Figure 3).

### Expression Analysis of the *Csa4G637110* and Other Related Genes

**FIGURE 5:**
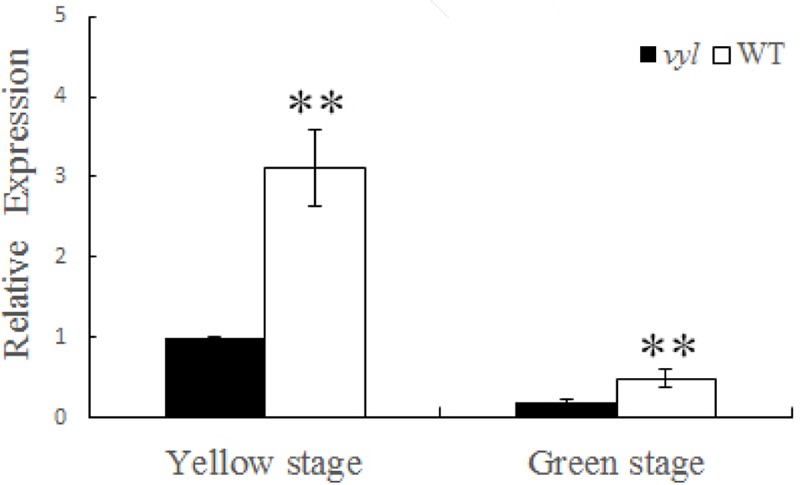
Relative expression of *Cs4G637110* gene at different leaf color stages (yellow and green stage) of WT and *vyl* mutant by qRT-PCR. Data were displayed using the *CsActin* gene as an internal control with three biological and three technical replicates. Values are the mean ± SD. ^∗∗^*P* < 0.01.

The expression level of *Csa4G637110* gene was measured at different leaf color stages (yellow and green stages) from *vyl* mutant and WT to analyze the relationship between expression patterns of *Csa4G637110* gene and the change of leaf color. According to qRT-PCR results, at the yellowing leaf stage of *vyl* mutant, the expression level of this gene in WT was significantly higher than that in *vyl* mutant leaves. Subsequently, the high expression level in WT decreased as the leaves aged and finally was equal to that in the *vyl* mutant (**Figure 5**).

**FIGURE 6:**
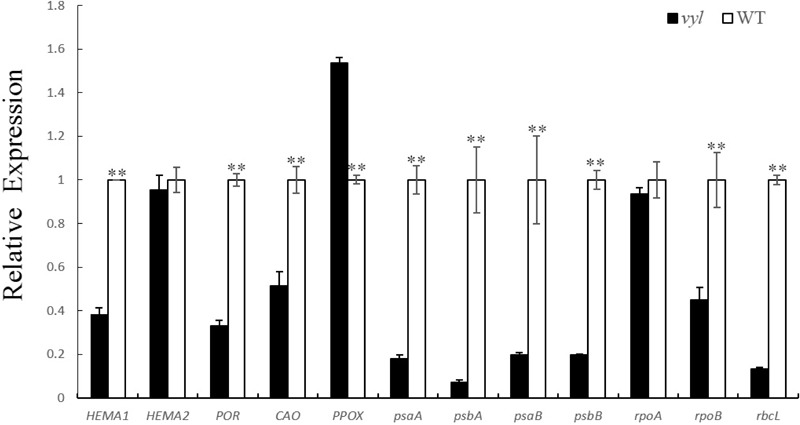
Expression evaluation of genes associated with Chl biosynthesis and photosynthesis through qRT-PCR. The L1 Leaves of *vyl* and WT were sampled. *CsActin* was used as the internal control. The transcript levels of all tested genes in wild type (WT) were set to 1.0. Values are the mean ± SD with three biological and three technical replicates. ^∗∗^*P* < 0.01.

The expression of the transcription of 12 genes involved in Chl biosynthesis and photosynthesis were analyze at seeding stage. The result was showed in **Figure 6**. There genes involved in Chl biosynthesis were down-regulated in *vyl*, including *HEMA1* (glutamyl-tRNA reductase), *POR* (NADPH-dependent protochlorophyllide oxidoreductase) and *CAO* (Chlorophyllide a oxygenase). In contrast, compared with the WT, the expression level of *PPOX* (protoporphyrinogen oxidase) was significantly increased in the *vyl* mutant. It is worthwhile to note that most of the expression levels of chloroplast genome genes were reduced in the *vyl* mutant, including *psaA, psaB, psbA*, and *psbB* (subunit genes of PS I and PS II core complex), *rbcL* (rubisco large subunit gene) and *rpoB* (plastid RNA polymerase gene). There was no obvious difference in the expression of *HEMA2* and *rpoA* (plastid RNA polymerase alpha subunit). The down-regulated expression levels of these genes implied that the mutant gene might involve in the Chl biosynthesis and photosynthesis pathway.

## Discussion

The main sources of leaf color mutants are natural mutagenesis or artificially induced mutagenesis, and most of leaf color mutants are controlled by recessive genes and occur at seedling stage (Pierce and Wehner, 1990). Some chlorotic types gradually turn green during the later leaf growth stage (Austin and Webber, 2005; Li et al., 2014), while some remained chlorosis in the whole development process which are usually lethal (Zhang et al., 2013). The virescent leaf color mutant used in this article was selected from M_2_ generation of ‘Changchunmici’ mutant library, which was caused by EMS mutagenesis. This virescent yellow leaf was regulated by a single recessive gene verified by genetic analysis. This *vyl* mutant showed yellow young leaf with obvious green leaf veins (**Figure 1B**), which is different with previous cucumber leaf color mutant *v* (Pierce and Wehner, 1990) and *v-1* (Miao et al., 2016). Also, different from the whole-life leaf color mutant (Gao et al., 2016), the leaf color of this *vyl* mutant gradually turned green as the leaves age, and the plant can develop, blossom and yield fruit normally. The changes of leaf color in *vyl* plant from young leaf to mature leaf were accompanied by changes in chlorophyll content and photosynthetic capacity (**Figure 3** and **Table 1**). Furthermore, we also verified that it was a light-sensitive mutation which showed virescent leaf only under strong light condition (**Figure 2**).

The mechanism of leaf chlorotic mutation is complicated and can generally be summarized into two possibilities. The first one is that the mutation of chlorophyll biosynthesis or degradation related gene directly leads to the change of pigment content in leaves, like *OsCHLI* and *OsCHLD* mutants (Zhang et al., 2006); the second possibility is that the mutation of chloroplast developmental genes directly leads to incomplete chloroplast development, therefore indirectly affects chlorophyll synthesis, like *ASL2* (Lin et al., 2015), *CSP41* (Mei et al., 2017) mutant gene in rice. In cucumber, the chlorophyll-deficient material, C528, has a golden leaf color throughout the whole life and chlorophyll content is lower in mutant than that in wild type. The corresponding gene, *CsChlI*, which encodes a CHLI subunit of cucumber Mg-chelatase, was cloned (Gao et al., 2016). Miao et al. (2016) used a virescent leaf color material (9110Gt) that shows incomplete chloroplast development, to clone a chloroplast development related gene, *CsaCNGCs*, which encodes a cyclic-nucleotide-gated ion channel protein. In this study, the chloroplast structure and chlorophyll content of the mutant were different from the wild type at yellowing stage. The grana stacks appeared sparse and irregular in shape, the thylakoid expanded largely, and assimilated products were less than in wild type (**Figure 3A**). The pigment of plant leaves was mainly located on the thylakoid of chloroplast. The chloroplast structure of the chlorotic mutant was damaged during chloroplast formation, which resulted in the abnormal development of thylakoid, eventually leading to decreased chlorophyll content compared to wild type (Motohashi et al., 2003). As the leaves were aging, the lamellar structure of grana stacks increased and the shape became regular. Chloroplast also gradually developed completely, the pigment levels were improved and leaves turned green similar to the wild type (**Figures 3B–D**). The delayed development of chloroplast is similar to that of 9110Gt (Miao et al., 2016), therefore, it can be speculated that the cause of yellowing young leaf is a result of damaged chloroplast structure.

For genetic mapping of *CsVYL* locus, chromosome walking combined with BSA and re-sequencing technology were adopted. In recent years, a number of phenotype-related genes have been cloned using the same strategy in cucumber (Li et al., 2013; Yang et al., 2014; Zhou et al., 2015). As a gene mapping method, it can quickly and accurately delineate target genes on an approximate position (Michelmore et al., 1991). Using two pools, 4 polymorphic SSR markers were screened from 450 SSR evenly distributed on 7 cucumber chromosomes. Then genotyping 80 plants of F_2_ population using the 4 markers allowed rapidly mapping the mutant gene in a 3.8 cM interval of chromosome 4. Next sequencing technology provides a powerful and cost-effective tool for exploring polymorphic markers during fine mapping (Liu et al., 2016). In this study, the parents (*vyl* and ‘Hazerd’) were re-sequenced to develop Indel and SNP markers in the target region, and finally the candidate gene was fine mapped in an 86.3 kb interval of chromosome 4, flanking by Indel53 and Indel56.

A total of 11 genes were detected in the 86.3 kb region. Through sequence alignment, a non-synonymous mutation leading to an amino acid sequence changes was found in *Csa4G637110* coding region, suggesting that the mutation identified is responsible for the virescent-yellow leaf phenotype of *vyl* (Supplementary Figure 1). In addition, the function of this candidate gene was also verified by expression analysis. The transcription level of *Csa4G637110* in *vyl* was significantly lower than that of WT during the young leaf stage. This high expression in WT reduced significantly when leaves aged. The expression pattern is similar to that of *v*-1 gene in cucumber (Miao et al., 2016), where a single-nucleotide mutation results in reduced expression of *CsaCNGCs*, and causes the occurrence of leaf color change. In addition, qPCR analysis results showed that most genes involved in Chl biosynthesis and photosynthesis were down-regulated in the *vyl* mutant at seeding stage (**Figure 6**). These findings indicated the important function of mutant gene in chloroplast development of cucumber at seeding stage. These findings suggested that *Csa4G637110* is a plausible candidate of *CsVYL*. However, complementary experiments are needed to confirm its reliability.

The cucumber DnaJ-like zinc finger protein and its orthologs have a similar zinc finger domain containing the CxxCxGxGx repeats structure (Supplementary Figure 2), which is commonly found in the DnaJ protein. However, different from the typical DnaJ protein, DnaJ-like zinc finger proteins does not contain specific J Domain of DnaJ proteins and G/F domain structure, and Muñoz-Nortes et al. (2017) classified it as the type IV DnaJ protein. As a companion protein of HSP70 (heat shock protein 70), DnaJ protein and HSP70 are composed of the molecular chaperone that involved in many cellular processes, including protein folding, assembly, translocation and degradation of misfolded proteins (Georgopoulos, 1992; Hartl et al., 1994; Craig et al., 2006). Many biological processes require the participation of this molecular chaperon in Chloroplast, such as chloroplast development, photosynthesis, chloroplast protein transport (Vitha et al., 2003; Latijnhouwers et al., 2010; Kong et al., 2014). Recent studies have shown that DnaJ-like zinc finger proteins also have similar functions, and several mutants of these proteins resulted in different leaf color changes, for example, *lqy1* and *sco2-1* in Arabidopsis (Lu et al., 2011; Tanz et al., 2012), *orange* in cauliflower (Li et al., 2001), *psa2* in maize (Fristedt et al., 2014). It can be speculated that the DnaJ-like zinc finger protein also has the same function in chloroplast development in cucumber. However, the regulatory mechanisms are still needed to be further researched.

## Author Contributions

QL and JC conceived the research and designed the experiments. MS and JW performed the research. MS, QW, JW, and QL analyzed the data and wrote the manuscript. XQ participated in genome re-sequencing analysis. WF, XL, FC and KY were involved in phenotypic selection, DNA extraction, and mapping work. LZ, JL, and XY provided valuable experimental methods. All authors read and approved the final manuscript.

## Conflict of Interest Statement

The authors declare that the research was conducted in the absence of any commercial or financial relationships that could be construed as a potential conflict of interest.

## Footnotes

1 http://cucumber.genomics.org.cn

2 http://linux1.softberry.com/berry.phtml

3 http://cmb.bnu.edu.cn/Cucumis_sativus_v20/

4 http://blast.ncbi.nlm.nih.gov/Blast.cgi
