# Supplementary material for: Fine Mapping of CsVYL, Conferring Virescent Leaf Through the Regulation of Chloroplast Development in Cucumber
Source: Front Plant Sci. 2018 Apr 6;9:432. doi: 10.3389/fpls.2018.00432 (PMC5897749; doi:10.3389/fpls.2018.00432)

Supplementary Figure 1 DNA sequence alignment of *Csa4G637110* among among 72 lines of the natural cucumber population. 9930, ccmc and *vyl*. The SNP mutant site are marked with red boxes.

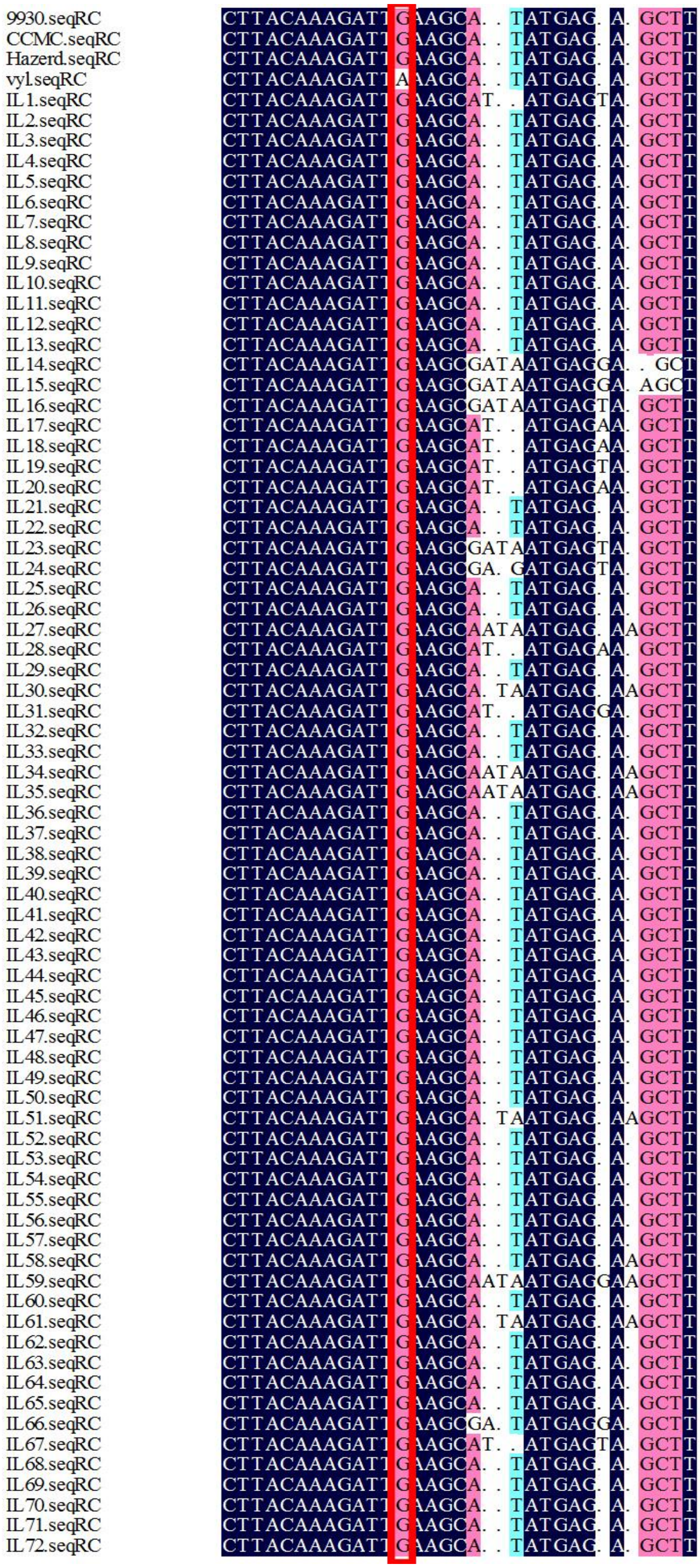

Supplement: Supplementary file 5 [file Image_1.PDF]
