# Supplementary material for: Fine Mapping of CsVYL, Conferring Virescent Leaf Through the Regulation of Chloroplast Development in Cucumber
Source: Front Plant Sci. 2018 Apr 6;9:432. doi: 10.3389/fpls.2018.00432 (PMC5897749; doi:10.3389/fpls.2018.00432)
Supplement: Supplementary file 6 [file Image_2.PDF]

Supplementary Figure 2 cDNA sequence alignment of *Csa4G637110* originated from 3 cucumbers as follow: 9930, ccmc and *vyl*.

```
vyl     ATGTCTAGCGGAACAGTCCGTCTGGTGAAGAAATTGAAGAAATTCGCTGACTTTCACTACAAGGTTTTCACTAGACGATATGGGCAGCAAGTCATTGACATCCTCGACCTTCCTCTTAAGGTGGTTTTATCCCCTTTCAC   140
ccmc    ATGTCTAGCGGAACAGTCCGTCTGGTGAAGAAATTGAAGAAATTCGCTGACTTTCACTACAAGGTTTTCACTAGACGATATGGGCAGCAAGTCATTGACATCCTCGACCTTCCTCTTAAGGTGGTTTTATCCCCTTTCAC   140
9930    ATGTCTAGCGGAACAGTCCGTCTGGTGAAGAAATTGAAGAAATTCGCTGACTTTCACTACAAGGTTTTCACTAGACGATATGGGCAGCAAGTCATTGACATCCTCGACCTTCCTCTTAAGGTGGTTTTATCCCCTTTCAC   140
Consensatgtctagcggaacagtccgtctggtgaagaaattgaagaaattcgctgactttcactacaaggttttcactagacgatatgggcagcaagtcattgacatcctcgaccttcctcttaaggtggttttatcccctttcac

vyl     CCTTGTTTTCGACATTGCCGGCTCAGCCCCTCGTGGCTTTGGTGTCCCCGAGCTAATTTCCAAGCTCTCATATGCTTTAATCTTTGCTATTGCTACATTTGGGACTTATGATATTGCGCTAGACCTAGGAAGGAAAGTTA   280
ccmc    CCTTGTTTTCGACATTGCCGGCTCAGCCCCTCGTGGCTTTGGTGTCCCCGAGCTAATTTCCAAGCTCTCATATGCTTCAATCTTTGCTATTGCTACATTTGGGACTTATGATATTGCGCTAGACCTAGGAAGGAAAGTTA   280
9930    CCTTGTTTTCGACATTGCCGGCTCAGCCCCTCGTGGCTTTGGTGTCCCCGAGCTAATTTCCAAGCTCTCATATGCTTCAATCTTTGCTATTGCTACATTTGGGACTTATGATATTGCGCTAGACCTAGGAAGGAAAGTTA   280
Consenscctt gttttcgacattgccggctcagcccctcgtggctttggtgtccccgagctaatttccaagctctcatatgctt aatctttgctattgctacatttgggacttatgatattgcgctagacctaggaaggaaagtta

vyl     TATGTCAAAGGCAGTGTCAAACCTGCAATGGATGGCAAGCCATGCGGTGTACTAAGTGCAGAGGGTCAGGGATGGTGAACTACCAAGTGAAGAACTATGTGTTGAGAAGTGGAGAGAAGCCAACACCAGAAAGTATTGCA   420
ccmc    TATGTCAAAGGCAGTGTCAAACCTGCAATGGATGGCAAGCCATGCGGTGTACTAAGTGCAGAGGGTCAGGGATGGTGAACTACCAAGTGAAGAACTATGTGTTGAGAAGTGGAGAGAAGCCAACACCAGAAAGTATTGCA   420
9930    TATGTCAAAGGCAGTGTCAAACCTGCAATGGATGGCAAGCCATGCGGTGTACTAAGTGCAGAGGGTCAGGGATGGTGAACTACCAAGTGAAGAACTATGTGTTGAGAAGTGGAGAGAAGCCAACACCAGAAAGTATTGCA   420
Consenstatgtcaaaggcagtgtcaaacctgcaatggatggcaagccatgcggtgtactaagtgcagagggtcagggatggtgaactaccaagtgaagaactatgtgttgagaagtggagagaagccaacaccagaaagtattgca

vyl     GATGCCATTGTAGAGAATCGGGCTGAGTTGATTCACCTTCCTTCCAGTTTGGATCTTCATACACCATTGCCATCCAAAGATTGCCCAACATGTGATGGAACAGGAGTGATGTGCTGCCCTGAATGCAAGAATAAATTGCC   560
ccmc    GATGCCATTGTAGAGAATCGGGCTGAGTTGATTCACCTTCCTTCCAGTTTGGATCTTCATACACCATTGCCATCCAAAGATTGCCCAACATGTGATGGAACAGGAGTGATGTGCTGCCCTGAATGCAAGAATAAATTGCC   560
9930    GATGCCATTGTAGAGAATCGGGCTGAGTTGATTCACCTTCCTTCCAGTTTGGATCTTCATACACCATTGCCATCCAAAGATTGCCCAACATGTGATGGAACAGGAGTGATGTGCTGCCCTGAATGCAAGAATAAATTGCC   560
Consensgatgccattgtagagaatcgggctgagttgattcaccttccttccagtttggatcttcatacaccattgccatccaaagattgcccaacatgtgatggaacaggagtgatgtgctgccctgaatgcaagaataaattgcc

vyl     ACTGAGAATCTCAGCAGACGATATCATGGAACCACCTTGGAAAGCGTATAATGTTATGAAAAAGATGGAATATCCATATGAGCATTTAGCTCATAGTATGAAGGACCCCAGCATTGCCGCATTTTGGTTACTTACTTTCC   700
ccmc    ACTGAGAATCTCAGCAGACGATATCATGGAACCACCTTGGAAAGCGTATAATGTTATGAAAAAGATGGAATATCCATATGAGCATTTAGCTCATAGTATGAAGGACCCCAGCATTGCCGCATTTTGGTTACTTACTTTCC   700
9930    ACTGAGAATCTCAGCAGACGATATCATGGAACCACCTTGGAAAGCGTATAATGTTATGAAAAAGATGGAATATCCATATGAGCATTTAGCTCATAGTATGAAGGACCCCAGCATTGCCGCATTTTGGTTACTTACTTTCC   700
Consensactgagaatctcagcagacgatatcatggaaccaccttggaaagcgtataatgttatgaaaaagatggaatatccatatgagcatttagctcatagtatgaaggaccccagcattgccgcattttggttacttactttcc

vyl     CTCAAATTGTTGGTGGATTCAACTTTGATGAAGATGTCAAGCAAAAGATTTGGTGGCAGTACAAGGAATCCATGCGATATGATCAACTCAGAGATGCTGTTGCTGAGCGGAAACCTGGATGGGAGTACTTACAAAAAGCC   840
ccmc    CTCAAATTGTTGGTGGATTCAACTTTGATGAAGATGTCAAGCAAAAGATTTGGTGGCAGTACAAGGAATCCATGCGATATGATCAACTCAGAGATGCTGTTGCTGAGCGGAAACCTGGATGGGAGTACTTACAAAAAGCC   840
9930    CTCAAATTGTTGGTGGATTCAACTTTGATGAAGATGTCAAGCAAAAGATTTGGTGGCAGTACAAGGAATCCATGCGATATGATCAACTCAGAGATGCTGTTGCTGAGCGGAAACCTGGATGGGAGTACTTACAAAAAGCC   840
Consensctcaaattgttggtggattcaactttgatgaagatgtcaagcaaaagatttggtggcagtacaaggaatccatgcgatatgatcaactcagagatgctgttgctgagcggaaacctggatgggagtacttacaaaaagcc

vyl     TTAATTTCCCTAGACCCTGTTCGAGCCAGGGATGATCCTGTGGTCGTGAAAAACATTCCTTACTTTAAGGCCAAGAAAGCACTTGAGGCAGAAGTGATAAAGCTTGATCCTCCACCACGGCCGCAAAATTGGGGTGAGTT   980
ccmc    TTAATTTCCCTAGACCCTGTTCGAGCCAGGGATGATCCTGTGGTCGTGAAAAACATTCCTTACTTTAAGGCCAAGAAAGCACTTGAGGCAGAAGTGATAAAGCTTGATCCTCCACCACGGCCGCAAAATTGGGGTGAGTT   980
9930    TTAATTTCCCTAGACCCTGTTCGAGCCAGGGATGATCCTGTGGTCGTGAAAAACATTCCTTACTTTAAGGCCAAGAAAGCACTTGAGGCAGAAGTGATAAAGCTTGATCCTCCACCACGGCCGCAAAATTGGGGTGAGTT   980
Consensttaatttccctagaccctgttcgagccagggatgatcctgtggtcgtgaaaaacattccttactttaaggccaagaaagcacttgaggcagaagtgataaagcttgatcctccaccacggccgcaaaattggggtgagtt

vyl     GGACCTTCCACTCAATTCATCTTCTTGGAGTCAGGATGATCTTAAAGACCCAGGAAAATTTAATGAAATGACTGTACTTCTAAATGCCCAAAGAGAAATTGCTGATAAAATCTTGGATGCACAGTGGGAAACAAAATGGC  1120
ccmc    GGACCTTCCACTCAATTCATCTTCTTGGAGTCAGGATGATCTTAAAGACCCAGGAAAATTTAATGAAATGACTGTACTTCTAAATGCCCAAAGAGAAATTGCTGATAAAATCTTGGATGCACAGTGGGAAACAAAATGGC  1120
9930    GGACCTTCCACTCAATTCATCTTCTTGGAGTCAGGATGATCTTAAAGACCCAGGAAAATTTAATGAAATGACTGTACTTCTAAATGCCCAAAGAGAAATTGCTGATAAAATCTTGGATGCACAGTGGGAAACAAAATGGC  1120
Consensggaccttccactcaattcatcttcttggagtcaggatgatcttaaagacccaggaaaatttaatgaaatgactgtacttctaaatgcccaaagagaaattgctgataaaatcttggatgcacagtgggaaacaaaatggc

vyl     GGCAGGAAAAGTTGAATGAGTTGTTGGAGGAAAAGCTGCGGCCTTACGTTACAAGTGCAAACAGTCATGTCCTTACAGCGCCAATTGTGTTGAAGTCAAAGGACCCGGAACAGAAGAGAAACCGACGGCGATGGTTTTGG  1260
ccmc    GGCAGGAAAAGTTGAATGAGTTGTTGGAGGAAAAGCTGCGGCCTTACGTTACAAGTGCAAACAGTCATGTCCTTACAGCGCCAATTGTGTTGAAGTCAAAGGACCCGGAACAGAAGAGAAACCGACGGCGATGGTTTTGG  1260
9930    GGCAGGAAAAGTTGAATGAGTTGTTGGAGGAAAAGCTGCGGCCTTACGTTACAAGTGCAAACAGTCATGTCCTTACAGCGCCAATTGTGTTGAAGTCAAAGGACCCGGAACAGAAGAGAAACCGACGGCGATGGTTTTGG  1260
Consensggcaggaaaagttgaatgagttgttggaggaaaagctgcggccttacgttacaagtgcaaacagtcatgtccttacagcgccaattgtgttgaagtcaaaggacccggaacagaagagaaaccgacggcgatggttttgg

vyl     TTCTTTTGA  1269
ccmc    TTCTTTTGA  1269
9930    TTCTTTTGA  1269
Consensttcttttga
```

.
