# Supplementary material for: Fine Mapping of CsVYL, Conferring Virescent Leaf Through the Regulation of Chloroplast Development in Cucumber
Source: Front Plant Sci. 2018 Apr 6;9:432. doi: 10.3389/fpls.2018.00432 (PMC5897749; doi:10.3389/fpls.2018.00432)

Supplementary Figure 3 Sequence alignment of the DnaJ-like zinc finger protein from 10 CsVYL's homologous. The DnaJ-like CR domain are highlighted in blue underline. The four zinc binding motifs are shaded green. Plant species used for the multiple sequence alignment: Cs, *Cucumis sativus* (XP_004141219.1); Cm, *Cucumis melo* (XP_008452442.1); Pp, *Prunus persica* (ONI12706.1); Pa, *Prunus avium* (XP_021801691.1); Jr, *Juglans regia* (XP_018850702.1); Jc, *Jatropha curcas* (XP_012076623.1); Pe, *Populus euphratica* (XP_011029493.1); Hu, *Herrania umbratica* (XP_021288076.1); At, *Arabidopsis thaliana* (OAO89767.1); Mc, *Momordica charantia* (XP_022139600.1).

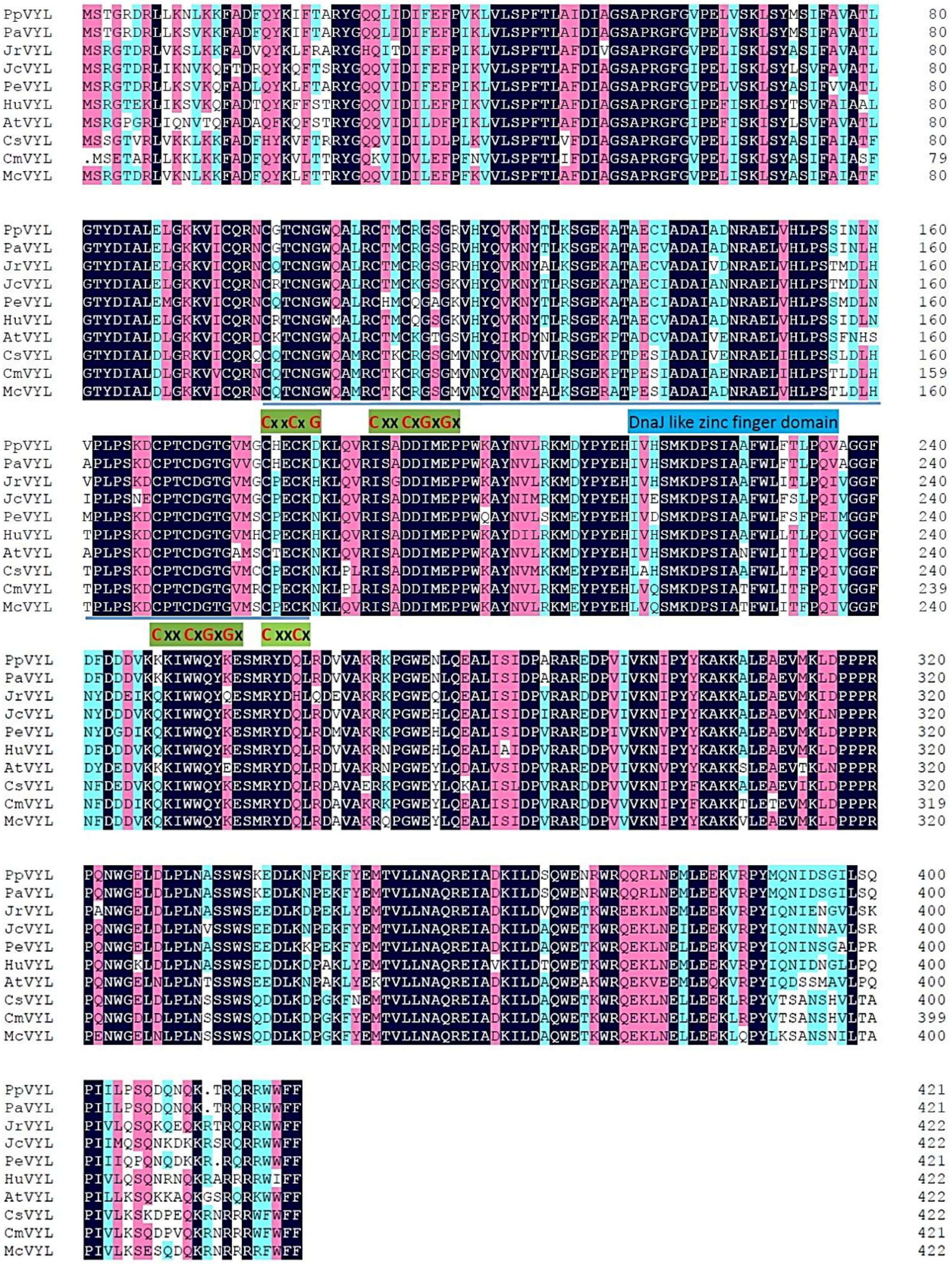

Supplement: Supplementary file 7 [file Image_3.PDF]
